# High resolution melting for mutation scanning of *TP53 *exons 5–8

**DOI:** 10.1186/1471-2407-7-168

**Published:** 2007-08-31

**Authors:** Michael Krypuy, Ahmed Ashour Ahmed, Dariush Etemadmoghadam, Sarah J Hyland, Australian Ovarian Cancer Study Group, Anna deFazio, Stephen B Fox, James D Brenton, David D Bowtell, Alexander Dobrovic

**Affiliations:** 1 Molecular Pathology Research and Development Laboratory, Department of Pathology, Peter MacCallum Cancer Centre, Locked Bag 1, A'Beckett St, Melbourne, Victoria 8006, Australia; 2 Functional Genomics of Drug Resistance Laboratory, Cancer Research UK Cambridge Research Institute, Li Ka Shing Centre, Robinson Way Cambridge CB2 0RE, UK; 3 Ian Potter Centre for Genomics and Predictive Medicine, Peter MacCallum Cancer Centre, Locked Bag 1, A'Beckett St, Melbourne, Victoria 8006, Australia; 4 Department of Biochemistry and Molecular Biology, University of Melbourne, Parkville, Victoria, 3010, Australia; 5 Hutchison/MRC Research Centre, University of Cambridge, Cambridge CB2 2XZ, UK; 6 Westmead Institute for Cancer Research, University of Sydney at Westmead Millennium Institute, Westmead Hospital, New South Wales 2145 Australia; 7 Department of Pathology, University of Melbourne, Parkville, Victoria, 3010, Australia

## Abstract

**Background:**

p53 is commonly inactivated by mutations in the DNA-binding domain in a wide range of cancers. As mutant p53 often influences response to therapy, effective and rapid methods to scan for mutations in *TP53 *are likely to be of clinical value. We therefore evaluated the use of high resolution melting (HRM) as a rapid mutation scanning tool for *TP53 *in tumour samples.

**Methods:**

We designed PCR amplicons for HRM mutation scanning of *TP53 *exons 5 to 8 and tested them with DNA from cell lines hemizygous or homozygous for known mutations. We assessed the sensitivity of each PCR amplicon using dilutions of cell line DNA in normal wild-type DNA. We then performed a blinded assessment on ovarian tumour DNA samples that had been previously sequenced for mutations in *TP53 *to assess the sensitivity and positive predictive value of the HRM technique. We also performed HRM analysis on breast tumour DNA samples with unknown *TP53 *mutation status.

**Results:**

One cell line mutation was not readily observed when exon 5 was amplified. As exon 5 contained multiple melting domains, we divided the exon into two amplicons for further screening. Sequence changes were also introduced into some of the primers to improve the melting characteristics of the amplicon. Aberrant HRM curves indicative of *TP53 *mutations were observed for each of the samples in the ovarian tumour DNA panel. Comparison of the HRM results with the sequencing results revealed that each mutation was detected by HRM in the correct exon. For the breast tumour panel, we detected seven aberrant melt profiles by HRM and subsequent sequencing confirmed the presence of these and no other mutations in the predicted exons.

**Conclusion:**

HRM is an effective technique for simple and rapid scanning of *TP53 *mutations that can markedly reduce the amount of sequencing required in mutational studies of *TP53*.

## Background

p53 is a tumour suppressor that plays a major role in regulating the cellular response to environmental and genotoxic stress, through cell cycle inhibition and promotion of programmed cell death or senescence [1-4]. There are a variety of stresses that have been shown to activate p53 including DNA damage, cell-cycle aberrations, hypoxia, and aberrant growth signals resulting from expression of oncogenes [5-10]. Moreover, a wide body of work has established the role of p53 in response to DNA damage [5-8].

It has been estimated that the gene encoding p53 (*TP53*) is mutated in more than 50% of human cancers [9]. It appears that inactivation of p53, by mutation or by other means, is highly advantageous and perhaps an absolute requirement for cancer progression (reviewed in Soussi, 2007). The prevalence of p53 mutations is highest in ovarian cancer (48.3%), followed by colorectal cancer (43.6%), oesophageal cancer (42.6%), head and neck cancer (41.5%), and lung cancer (38.4%) [10].

In ovarian cancer, mutation of the *TP53 *gene appears to play an important role in serous carcinogenesis, where *TP53 *mutations are present in approximately 50% of high-grade serous carcinomas but rare in serous borderline tumours and low-grade serous carcinoma [11,12]. Mutations in *TP53 *are also common in ovarian and breast tumours that are *BRCA1*-related [13,14].

Mutations can occur throughout *TP53*. However there are also "hot spots" that account for many mutations [10,15]. In the majority of cancers, p53 dysfunction is caused through a direct mutation within the DNA-binding domain of the gene [9]. Moreover mutations in exons 5 to 8 of *TP53 *comprise 94.2% of all somatic mutations in the IARC database, version R11 [10].

Different *TP53 *mutations appear to have different consequences [16-18]. Work on the common p53 mutants, R273H and R175H, using knock-in *in vivo *mice models showed that each mutation was responsible for distinct tumour patterns and characteristics, compared to mice with complete loss of one p53 allele [19,20]. These studies demonstrate that p53 mutant alleles may have an oncogenic potential beyond the simple loss of p53 function. This is also borne out by clinical studies in human cancer where certain types of *TP53 *mutations are associated with a poorer prognosis compared to other types of mutations. For example, studies have shown that *TP53 *mutations affecting the zinc binding domains or missense mutations in the DNA binding domain correlate with poorer prognosis in breast cancer [16,21].

Overabundance of p53 by immunohistochemistry has been commonly used as a surrogate marker for *TP53 *mutation in a wide range of cancers and although the method is cost effective, it does not have the required sensitivity to predict *TP53 *mutations [12,22]. Dideoxy sequencing remains the gold standard method to detect these mutations. However, it has the disadvantages of high cost in terms of labour and reagents. Sequencing is used most effectively as a confirmatory method after pre-screening with a mutation scanning technique for samples that ideally have had enrichment of the tumour component before DNA extraction.

High resolution melting (HRM) is a mutation scanning technique that monitors the progressive change in fluorescence caused by the release of an intercalating DNA dye from a DNA duplex as it is denatured by marginal increases in temperature [23]. It is an in-tube method requiring the inclusion of an saturating intercalating dye in the PCR mix and the addition of a high resolution melt step after PCR. The technique has already been employed to scan for somatic mutations in the *KIT*, *BRAF*, *EGFR, ERBB2*, and *KRAS genes *[24-27]. In this study, we show that high resolution melting is a highly effective scanning technique for mutations in *TP53*.

## Methods

### Study samples

Ovarian tumour samples were obtained from the Westmead Gynaecological Oncology Tissue Bank (WGOTB) and the Australian Ovarian Cancer Study (AOCS) [28], a multi-centre study established to recruit women with a suspected diagnosis of primary epithelial ovarian cancer, including peritoneal and fallopian tube cancers, from across Australia. Collection of clinical data and biological materials was subject to informed consent by the patient. Tissue collection (WGOTB) was approved by the Western Sydney Area Health Service Human Research Ethics Committee. Tissue collection (AOCS) was approved by the appropriate Ethics of Human Research Committees at the Peter MacCallum Cancer Centre, Queensland Institute of Medical Research, University of Melbourne and all participating hospitals. An experienced pathologist reviewed each sample. Tumour cells were enriched by needle-dissection of serial frozen tissue sections with reference to the flanking H&E stained sections. Regions within sections that had greater than 80% tumour material were selected for needle-dissection. For tissue with a high tumour component (>80%), whole tumour material was used for DNA extraction. Genomic DNA was extracted using the DNeasy kit (Qiagen, Hilden, Germany) according to the manufacturer's protocol. Ovarian cancer samples with typical *TP53 *mutations were selected to test sensitivity and positive predictive value of the HRM methodology. The breast tumour samples were collected from patients who underwent surgery at the John Radcliffe Hospital, Oxford, UK, according to the institute's ethical guidelines (approval number C02.216). The tumours underwent pathological review and genomic DNA was extracted from fresh frozen tissue.

### HRM assay conditions

**Figure 1:**
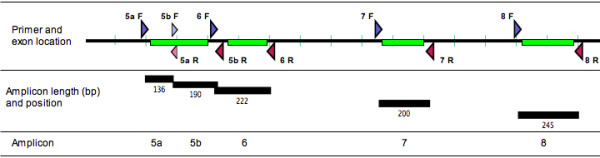
**Schematic representation of amplicons for the *TP53 *HRM assay**. Forward primers are in blue and reverse are in red. Exons 5 to 8 are represented by the green bars and the amplicon length and name are listed in the lower panels. Two amplicons were designed to span *TP53 *exon 5.

The DNA samples were further diluted with PCR grade water to a concentration of 0.5 ng/μL for use in PCR. Normal control DNA and cell line DNA was extracted using a salting out method [29]. Primers were designed to flank the coding regions and to be annealed at 60°C using Primer Express software to calculate the "Tm" (Applied Biosystems, Foster City, CA). The final optimal reaction conditions were empirically determined. Final primer sequences and the PCR annealing conditions are listed in Table 1. A schematic representation of *TP53 *HRM assay exons 5 to 8 is shown in Figure 1. The reaction mixture used HotStarTaq (Qiagen, Hilden Germany) and consisted of 2.5 ng of genomic DNA, 1× PCR buffer, 2.5 mM MgCl_2_, 200 nM of each primer, 200 μM of dNTPs, 5 μM of SYTO 9 (Invitrogen, Carlsbad, USA), 0.5 U of HotStarTaq polymerase and PCR grade water in a volume of 20 μL. All PCR reactions were performed in triplicate. PCR cycling and HRM analysis was performed on the Rotor-Gene™ 6000 (Corbett Research, Mortlake, New South Wales, Australia). The PCR cycling conditions were as follows; one cycle of 95°C for 15 minutes; 50 cycles of 95°C for 10 seconds, annealing conditions (see Table 1) for 5 seconds, 72°C for 20 seconds; one cycle of 95°C for 1 second, 72°C for 90 seconds and a HRM step from 72 to 95°C rising at 0.1°C per second.

**Table 1:** *TP53 *HRM primers and PCR annealing temperature conditions

**Exon**	**Primer name**	**Sequence**	**Annealing conditions**
5a	*TP53*_Exon5a_F	CAACTCTGTCTCCTTCCTCTTCCTAC	65–60°C touchdown 0.5°C/cycle for 10 cycles
	*TP53*_Exon5a_R	AGCCATGGCACGGACGCG	
5b	*TP53*_Exon5b_F	CTCCTGCCCGGCACCCGC	65–60°C touchdown 0.5°C/cycle for 10 cycles
	*TP53*_Exon5b_R	CTAAGAGCAATCAGTGAGGAATCAGA	
6	*TP53*_Exon6_F	CAACCACCCTTAACCCCTCCT	68–58°C touchdown 1.0°C/cycle for 10 cycles
	*TP53*_Exon6_R	AGACGACAGGGCTGGTTGC	
7	*TP53*_Exon7_F	AGGCGCACTGGCCTCATC	68–58°C touchdown 1.0°C/cycle for 10 cycles
	*TP53*_Exon7_R	GAGGCTGGGGCACAGCA	
8	*TP53*_Exon8_F	GACCTGATTTCCTTACTGCCTCTTG	63.5–58.5°C touchdown 0.5°C/cycle for 10 cycles
	*TP53*_Exon8_R	AATCTGAGGCATAACTGCACCCTT	

Underlined nucleotides are introduced sequence

### Sequencing methods

#### Ovarian tumour DNA samples

*TP53 *exons 5 to 8 were amplified using primers covering the exons and exon-intron boundaries (see Table 2). Amplification reactions (50 μl) contained 10 ng of DNA, 10 pmoles of each primer and 1.5 U of Amplitaq Gold DNA polymerase (Applied Biosystems) according to manufacturer's instructions. The amplifications were done using a DNA Engine Tetrad, PTC-225 thermal cycler (Biorad, Hercules, CA). PCR products were purified using the NucleoFast 96 PCR plates from Macherey-Nagel using the manufacturer's protocol. Purified PCR products were sequenced using ABI Prism *BigDye *terminators v3.1 (Applied Biosystems) and an ABI 3100 genetic analyser (Applied Biosystems). The results were analysed using SeqScape v1.0 (Applied Biosystems). Sequencing reactions were performed in forward and reverse directions. All sequences were manually examined. All exons with a mutation were re-amplified and re-sequenced in two directions.

**Table 2:** *TP53 *sequencing primers and annealing temperature conditions

**Exon**	**Primer name**	**Sequence**	**Annealing temperature**
5	*TP53*_5F	CTCTGTCTCCTTCCTCTTCC	55°C
	*TP53*_5R	GCAATCAGTGAGGAATCAGAGG	
6	*TP53*_6F	AGATAGCGATGGTGAGCAGC	60°C
	*TP53*_6R	ACTGACAACCACCCTTAACC	
7	*TP53*_7F	CAGGTCTCCCCAAGGCGCAC	60°C
	*TP53*_7R	GCAAGCAGAGGCTGGGGCAC	
8	*TP53*_8F	GGAGTAGATGGAGCCTGGTT	58°C
	*TP53*_8R	GTGAATCTGAGGCATAACTG	

#### Breast tumour DNA samples

The HRM products were directly sequenced except that the *TP53*_Exon5a_F *and TP53*_Exon5b_R primers (Table 1) were used to amplify the entire *TP53 *exon 5 for sequencing using the HRM reaction mixture at the following conditions; one cycle of 95°C for 15 minutes; 50 cycles of 95°C for 10 seconds, 65°C to 60°C touchdown (10 cycles at 0.5°C per step) for 5 seconds, 72°C for 20 seconds. The PCR products were column purified using the PCR-M clean up kit (Viogene, Taipei, Taiwan) according to the manufacturer's instructions. The PCR products were eluted in a 50 μl volume, and 6 μl was treated with ExoSapIT (GE Healthcare, Buckinghamshire, England) according to the manufacturer's instructions. The purified PCR product was then used as template in cycle sequencing in both forward and reverse directions with the Big Dye Terminator v3.1 kit (Applied Biosystems). The reaction mix consisted of 1× terminator premix, 1× sequencing buffer, 667 nM primer and 3.5 μl of cleaned template in a 15 μl total volume. The reactions were run on a PTC-225 thermal cycler according to the following protocol; one cycle of 95°C for 1 minute; 25 cycles of 95°C for 10 seconds, annealing temperature (64°C for full exon 5 and exon 8, 68°C for exons 6 and 7) for 30 seconds, 72°C for 3 minutes. The sequencing reactions were ethanol precipitated and run on a 3100 Genetic Analyser. All sequences were manually examined using Sequencher™ 4.6 (Gene Codes Corporation, Ann Arbor, MI).

### HRM sensitivity testing

The cell lines used in this study are listed in Table 3. Cell line DNA was mixed with wild-type DNA in dilutions of 50%, 25%, 10% and 5% to determine the sensitivity of mutation detection of each amplicon. BT-20 and SKBR3 were used to test sensitivity of the 5a and 5b amplicons respectively. T47D and OVCAR-3 was used to detect the sensitivity of the exon 6 and 7 amplicons respectively. RPMI8226 was used to assess the sensitivity of the exon 8 amplicon. The cell line dilutions were assessed by several individuals in a blinded fashion where scoring depended on being able to confidently differentiate the mutant containing DNA dilutions from the wild-type DNA samples. T47D was used in additional mixing experiments where dilutions were made in wild-type DNA at 95%, 90% and 75% to assess the degree of heteroduplex formation at higher mutant DNA proportions.

**Table 3:** *TP53 *mutant cell lines used in study

**Sample ID**	**Mutation**	**Protein change**	**Codon**	**Exon**
BT-20	394A>C	K132Q	132	5
SKBR3	524G>A	R175H	175	5
T47D	580C>T	L194F	194	6
KG-1	673insATCTG	frameshift	225	6
OVCAR-3	743G>A	R248Q	248	7
SW480	818G>A	R273H	273	8
MDA-MB231	839G>A	R280K	280	8
RPMI8226	853G>A	E285K	285	8

## Results

### HRM assay optimisation

The HRM amplicons for *TP53 *exons 5 to 8 are illustrated in Figure 1 and the primer sequences listed in Table 1. Initially, amplicons for HRM analysis were designed to span each of *TP53 *exons 5 to 8. We then tested the mutation detection capability of each amplicon using a panel of cancer cell lines (BT-20, SKBR3, T47D, KG-1, OVCAR-3, SW480, MDA-MB231 RPMI8226) that were homozygous or hemizygous for known *TP53 *mutations.

**Figure 2:**
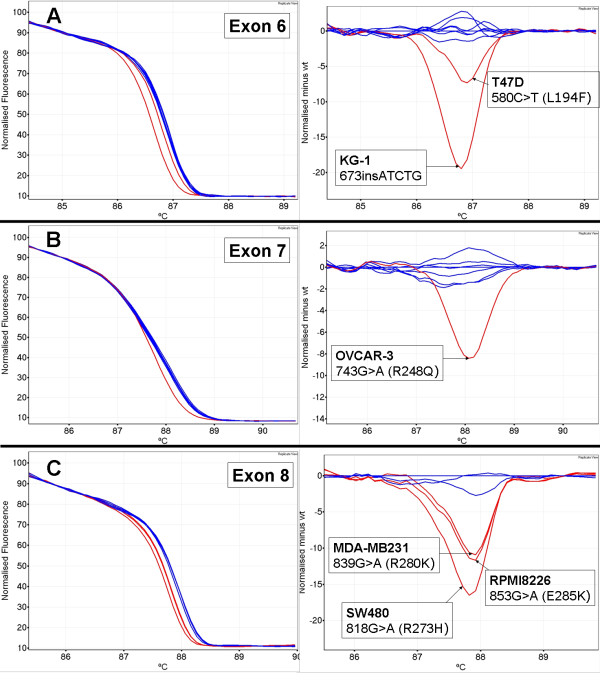
***TP53 *cell line mutations detected by HRM**. Cell line mutant samples are in red and wild-type samples are in blue. Normalised plots are on the left and the corresponding difference plots are on the right. Panel A - T47D 580C>T mutation in exon 6. Panel B - OVCAR-3 743G>A mutation in exon 7. Panel C - MDA-MB231 839G>A, SW480 818G>A, RPMI8226 853G>A mutations in exon 8.

Figure 2 shows normalised plots and difference plots of each cell line mutant compared to wild-type controls for amplicons for exons 6, 7 and 8. In most cases, the difference between mutant and wild-type patterns was clear. However, the T47D cell line (580C>T mutation, codon 194) had a melt profile that showed a subtle change in melting behaviour (estimated Tm difference of 0.1°C) compared to wild-type samples for exon 6 (Figure 2 panel A). It must be borne in mind, however, that the difficulty of detecting this change was due to only mutant sequence being present and that most clinical samples would have a mixture of normal cells with the resultant heteroduplexes considerably facilitating mutation detection.

**Figure 3:**
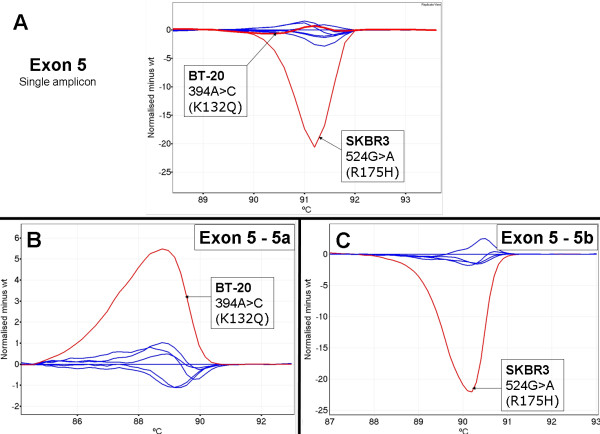
***TP53 *exon 5 amplicon redesign allowed detection of the BT-20 645A>C mutation**.  Cell line mutant samples are in red and wild-type samples are in blue. All plots shown in this figure are difference plots. Panel A; BT-20 394A>C and SKBR3 524G>A mutations with the original amplicon designed for exon 5. Panel B; BT-20 394A>C mutation is now detectable with the 5a amplicon. Panel C; SKBR3 524G>A mutation with the 5b amplicon.

We could not detect the 394A>C mutation in codon 132 of the BT-20 cell line with the initial amplicon we designed to span exon 5, although the 524G>A mutation in codon 175 of the SKBR3 cell line was detectable (Figure 3, panel A). Examination of the *TP53 *exon 5 sequence showed a GC rich region in the central region of the exon (position 451 to 477 from start codon, CCCCCGCCCGGCACCCGCGTCCGCGCC) that resulted in several melting domains within the amplicon. We hypothesized that the multiple melting domains were masking the presence of the mutation in BT-20. Subsequently, we designed 2 overlapping amplicons for exon 5, named 5a and 5b. This redesign relocated the GC rich regions to the end of each fragment.

As the first fragment of exon 5 still had a very GC rich domain at the 3' end and the second fragment still had a GC rich domain at the 5' end, we adopted the novel strategy of introducing sequence changes into the amplicons via the primers to promote more homogeneous melting characteristics as determined by the Poland program which simulates the melting of double stranded DNA [30]. Figure 3, panel B shows that the A to C mutation in BT-20 is readily detectable using the redesigned 5a amplicon.

### Sensitivity testing results using cell lines

**Figure 4:**
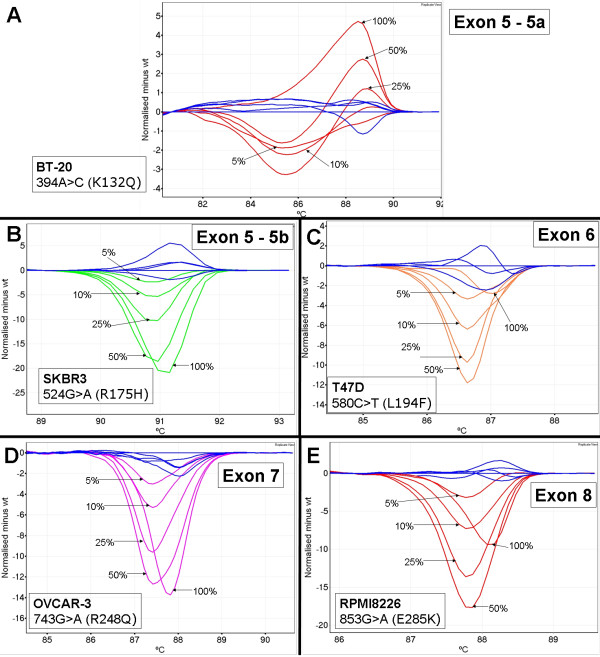
**Dilution of cell line mutant DNA to test sensitivity of *TP53 *HRM amplicons**. Wild-type samples are in blue and all plots shown are difference plots. Panel A: BT-20 dilution series. Panel B; SKBR3 dilution series. Panel C; T47D dilution series. Panel D; OVCAR-3 dilution series. Panel E; RPMI8226 dilution series.

We estimated the sensitivity of HRM for each amplicon with dilutions of *TP53 *mutant cell line DNA in wild-type DNA for the cell lines BT-20, SKBR3, T47D, OVCAR-3 and RPMI8226. Dilutions were made at 50%, 25%, 10% and 5% mutant DNA and the results were assessed by several individuals in a blinded fashion. Cell line DNA down to 5% was detected for each *TP53 *amplicon. Figure 4 shows the difference plots for each cell line dilution for each amplicon.

The T47D cell line was used to test the exon 6 amplicon. We could not readily distinguish the 100% T47D melt profile from the wild-type melt profiles. It appears that the minimal difference in melting behaviour between T47D and wild-type combined with spread of the wild-type melt profiles, contributed to the difficulty in scoring (Figure 4, panel C). Mixing experiments with T47D showed that at dilutions of 95%, 90% and 75%, there was sufficient heteroduplex formation to allow ready detection of the mutant sequence [see Additional file 1].

### Screening *TP53 *exons 5 to 8 in the ovarian tumour panel

**Figure 5:**
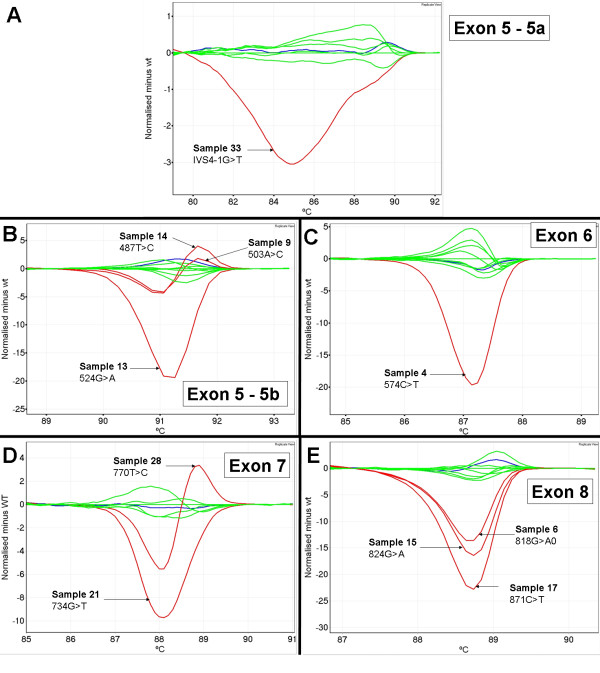
**Example of aberrant melt profiles observed for ovarian tumour samples**. Wild-type control samples are in blue. Wild-type patient samples are in green and mutant patient samples are in red. Panel A; mutation in patient 33 in exon 5 with amplicon 5a. Panel B; mutations in patients 9, 13 and 14 in exon 5 with amplicon 5b. Panel C; mutation in patient 4 in exon 6. Panel D; mutations in patients 21 and 28 in exon 7. Panel E; mutations in patients 6, 15 and 17 in exon 8.

We screened 20 ovarian tumour biopsy samples that had previously been sequenced for mutations in *TP53 *exons 5 to 8. Analysis of the HRM data was done blinded to the sequencing results. We observed the presence of aberrant melt profiles in all 20 samples. The majority of the melt profiles were clearly distinguishable on difference plots from wild-type by amplitude and/or by shape. Figure 5 shows examples of aberrant melt profiles for each amplicon.

Table 4 lists all the HRM and sequencing results for each of the ovarian tumour DNA samples. The sequencing results showed that there were *TP53 *mutations in all 20 samples (unknown to the HRM scorers at the time of the blinded analysis) and aberrant profiles by HRM were observed for each sample in the correct exons. There were 18 mutations that occurred with the coding region of *TP53 *and two changes present in the intronic sequence, one in intron 4–5 and the other in intron 5–6.

**Table 4:** Summary of HRM and sequencing results for the AOCS ovarian tumour panel

**Sample ID**	**HRM results**					**Sequencing results**			
	**Exon 5 5a**	**Exon 5 5b**	**Exon 6**	**Exon 7**	**Exon 8**	**Nomenclature**	**Protein change**	**Zygosity**	**Exon**

4	wt	wt	MUT	wt	wt	574C>T	Q192Stop	het	6
6	wt	wt	wt	wt	MUT	818G>A	R273H	het	8
9	wt	MUT	wt	wt	wt	503A>C	H168P	het	5
11	wt	wt	wt	MUT	wt	734G>A	G245D	het	7
13	wt	MUT	wt	wt	wt	524G>A	R175H	hom	5
14	wt	MUT	wt	wt	wt	487T>C	Y163H	het	5
15	wt	wt	?	wt	MUT	824G>A	C275Y	het	8
16	wt	wt	wt	MUT	wt	743G>A	R248Q	het	7
17	wt	wt	wt	wt	MUT	871C>T	R273C	het	8
19	wt	wt	wt	MUT	wt	713G>A	C238Y	het	7
21	wt	wt	?	MUT	wt	734G>T	G245V	-	7
22	Wt	wt	MUT	wt	wt	578A>G	H193R	-	6
25	Wt	MUT	wt	wt	wt	493C>T	Q165Stop	hom	5
27	Wt	wt	MUT	wt	wt	IVS5-2A>C	-	-	intron 5
28	Wt	wt	wt	MUT	wt	770T>C	L257P	het	7
33	MUT	wt	wt	wt	wt	IVS4-1G>T	-	-	intron 4
35	Wt	wt	wt	wt	MUT	818G>A	R273H	het	8
45	Wt	MUT	wt	wt	wt	499C>T	Q167Stop	het	5
48	Wt	wt	wt	MUT	wt	772G>A	E258K	het	7
59	Wt	MUT	wt	wt	wt	488A>G	Y163C	het	5

wt - wild type, MUT - Mutant, het – heterozygous, hom – homozygous, ? – unable to score

Samples that were mutation-negative for a particular exon were internal negative controls and all mutations detected by sequencing were detected by HRM. Each mutation was correctly identified in each exon giving 100% sensitivity for HRM within this sample set. We were uncertain about the mutation status of 2 samples in exon 6 and these proved to be negative by sequencing. This gave a positive predictive value of 91% for HRM within this sample set.

**Figure 6:**
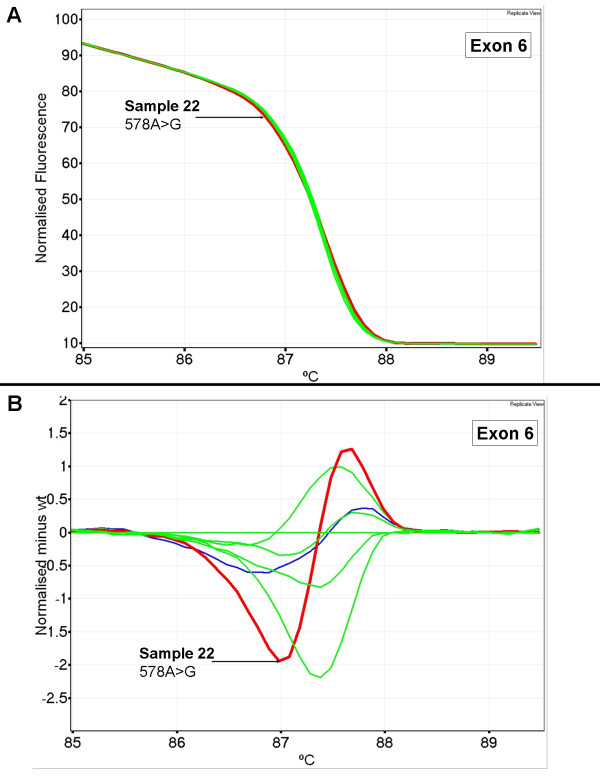
**Subtle change in melt profile observed for patient 22 in *TP53 *exon 6**. Wild-type control samples are in blue. Wild-type patient samples are in green and mutant patient samples are in red. Panel A; normalised plot of patient 22 compared to wild-type controls and wild-type ovarian tumour samples in exon 6. Panel B; difference plot of patient 22 compared to wild-type controls and wild-type ovarian tumour samples.

Figure 6 shows a normalised plot and a difference plot of *TP53 *exon 6 for patient 22. This sample had a subtle change in melting behaviour as seen with the slightly altered shape of the melt profile when compared to wild-type samples (Figure 6, panel A). The change in shape is more clearly seen with the difference plot with a prominent early melt, characteristic of heteroduplexes (Figure 6, panel B). The melt profile for patient 22 was close to the range of what would be classified as normal as the Tm difference was between it and the wild-type samples was minimal. Patient 22 had a 578A>G mutation identified by sequencing.

### Screening *TP53 *exons 5 to 8 in the breast tumour panel

**Figure 7:**
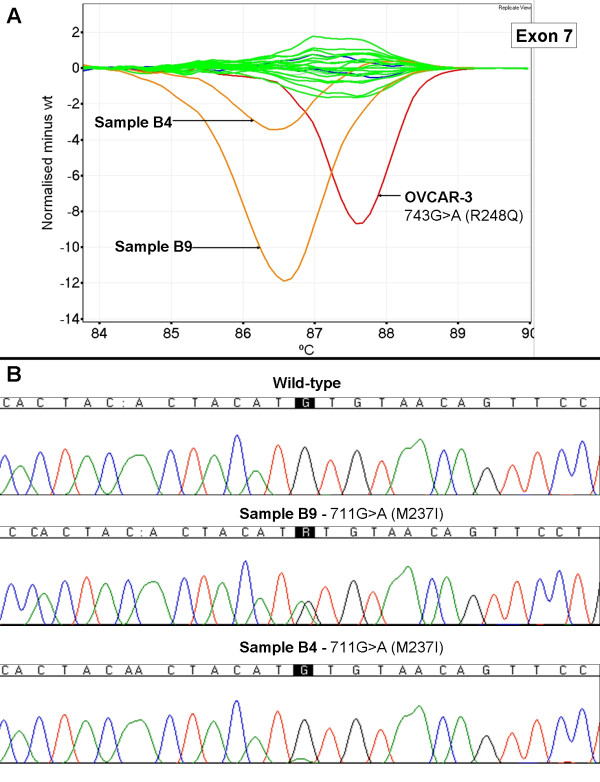
**HRM difference plot and sequencing data for breast tumour samples for TP53 exon 7**. Panel A; Difference plot of breast tumour samples for *TP53 *exon 7. Wild-type control samples are in blue, wild-type patient samples are in green, the cell line OVCAR-3 is in red and samples B4 and B9 are in orange. Panel B; Sequencing traces for wild-type, B4 and B9.

**Figure 8:**
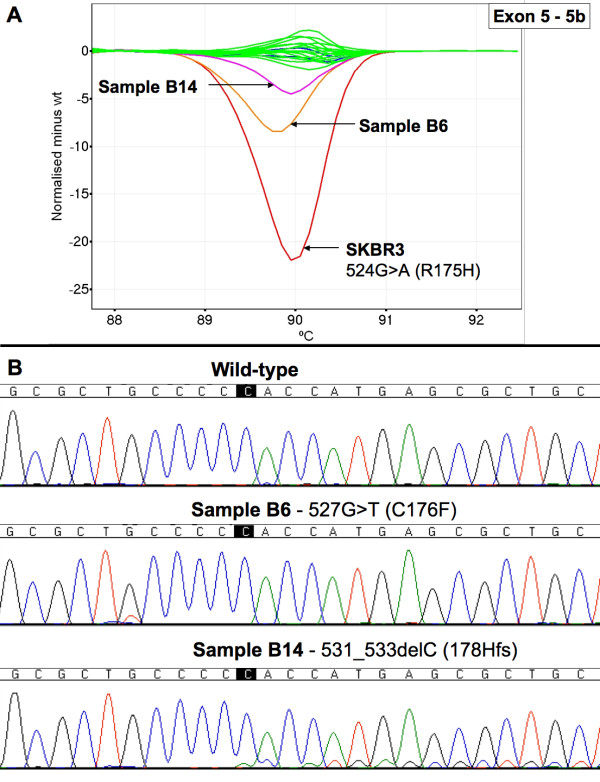
**HRM difference plot and sequencing data for breast tumour samples for *TP53 *exon 5, 5b amplicon**. Panel A; Difference plot of breast tumour samples for *TP53 *exon 5, amplicon 5b. Wild-type control samples are in blue, wild-type patient samples are in green, the cell line SKBR3 is in red, sample B6 is in orange and sample B14 is in pink. Panel B; Sequencing traces for wild-type, B6 and B14.

We screened 20 breast tumour biopsy samples with unknown *TP53 *mutation status using HRM. We observed the presence of aberrant melt profiles in 7 of the samples. No mutations were detected by sequencing in samples scored as wild-type by HRM. The majority of the melt profiles were clearly distinguishable on difference plots from wild-type by amplitude and/or by shape. Figure 7 shows examples of aberrant melt profiles for *TP53 *exon 7 for samples B4 and B9. The low peak height in the sequencing trace for the 711G>A mutation in sample B4 was reflective of the melt profile in HRM, where the shift was of a lower magnitude than sample B9. The data for samples B4 and B14 (Figure 8) indicated that mutations detectable by HRM were at the lower limit of detection by dideoxy sequencing.

Table 5 lists all the HRM and sequencing results for the breast tumour DNA samples. The sequencing results confirmed that there were *TP53 *mutations in all samples that had aberrant curves by HRM. There were 5 missense mutations and 2 null mutations.

**Table 5:** Summary of HRM and sequencing results for the breast tumour panel


**Sample ID**	**HRM results**					**Sequencing results**		
	**Exon 5 5a**	**Exon 5 5b**	**Exon 6**	**Exon 7**	**Exon 8**	**Nomenclature**	**Protein change**	**Exon**

B1	wt	wt	wt	wt	wt	NA	NA	NA
B2	wt	wt	wt	wt	wt	NA	NA	NA
B3	wt	MUT	wt	wt	wt	524G>A	R175H	5
B4	wt	wt	wt	MUT	wt	711G>A	M237I	7
B5	wt	wt	wt	wt	wt	NA	NA	NA
B6	wt	MUT	wt	wt	wt	527G>T	C176F	5
B7	wt	wt	wt	wt	wt	NA	NA	NA
B8	wt	wt	wt	wt	wt	NA	NA	NA
B9	wt	wt	wt	MUT	wt	711G>A	M237I	7
B10	wt	wt	wt	wt	wt	NA	NA	NA
B11	wt	wt	wt	wt	wt	NA	NA	NA
B12	wt	wt	wt	wt	wt	NA	NA	NA
B13	wt	wt	wt	wt	wt	NA	NA	NA

B14	wt	?	wt	wt	wt	531_533delC	frame shift, H178fs	5
B15	wt	wt	wt	wt	wt	NA	NA	NA
B16	wt	wt	MUT	wt	wt	614A>T	Y205F	6
B17	wt	wt	wt	wt	wt	NA	NA	NA
B18	wt	wt	wt	wt	wt	NA	NA	NA

B19	wt	MUT	wt	wt	wt	513_515delGinsAT	frame shift, V172fs	5

B20	wt	wt	wt	wt	wt	NA	NA	NA

wt - wild-type, MUT – Mutant, het – heterozygous, hom – homozygous, ? – unable to score, NA – not applicable

## Discussion

There have been numerous methods employed to detect *TP53 *mutations, each with its particular advantages and disadvantages. Scanning methodologies such as SSCP, gradient gel electrophoresis and denaturing high performance liquid chromatography (DHPLC) are advantageous because they significantly reduce the amount of sequencing that ultimately needs to be performed [31-36], streamlining the mutation detection process and making it more cost effective. The high resolution melting methodology presented here has the additional advantages over other scanning methodologies of increased simplicity and rapid turn-around-time, because it is performed directly after PCR amplification in the same tube. We chose exons 5 to 8 because over 94% of reported mutations occur in these regions [10].

Although the sensitivity is likely to be dependent on the particular mutation, the cell line testing results indicate a high sensitivity for HRM down to 5% mutant sequence. The sensitivity testing results also indicate that the overall variation of biological replicates of wild-type melt profiles will affect the ability to discriminate mutant samples from wild-type, which will ultimately affect the sensitivity of the technique.

Samples with pure mutant DNA could pose a problem for detection with HRM. For example, in cell lines containing only a mutant allele due to LOH of the remaining WT allele, in some cases (e.g. T47D) the resultant PCR product has minimal temperature difference compared to that amplified from wild-type DNA. However, this is a theoretical rather than a practical consideration as a proportion of wild-type DNA will be present in a tumour biopsy even with microdissection of the tissue specimen. Indeed, the addition of a small amount of wild-type DNA (5%) to T47D caused a sufficient heteroduplex effect to facilitate detection by HRM [see Additional file 1]. Addition of 5–10% wild type DNA should be considered for samples are known to be extremely pure, such as those that have been purified by laser-capture microdissection.

HRM analysis requires careful attention to the design of the amplicons as mutation detection is easier when there is a single melting domain. This is exemplified by exon 5 in which the 394A>C mutation in the 5' end of the exon was not initially observed under the conditions used. This was even the case when the cell line was diluted with normal DNA to create a heterozygous mutation (data not shown). We found that separating the amplicon into 2 domains allowed the detection of this mutation and all other mutations tested. We furthermore manipulated the primers, which encompassed a GC region between the 2 domains by introducing sequence changes into the primers, which promoted greater homogeneity in the melting behavior of the amplicon.

The ovarian tumour DNA panel was used to assess the sensitivity and positive predictive value of HRM for *TP53 *exons 5 to 8. The samples chosen were preselected for mutations in these exons but otherwise we performed a blinded analysis. There were a number of samples that had large shifts from the melt profiles of wild-type samples whereas others had changes that were more subtle. The aberrant curve produced for sample 22 in exon 6 appeared closer to a wild-type sample than a mutant because the profile existed within the spread for samples resembling wild type. However, the sample was classified as having an aberrant melt profile because of its conspicuously different shape, the earlier melting that is characteristic of heteroduplex formation, and because replicates gave the same pattern (Figure 6, panel B). The sequencing data showed that sample 22 had an A to G change at base 578. In this sample set, the HRM methodology had 100% sensitivity and 91% positive predictive value.

The screening of 20 breast tumour DNA samples with an unknown *TP53 *mutation status followed by sequencing of all the samples allowed us to further assess the sensitivity and positive predictive value of the methodology. Seven samples had aberrant melt profiles indicating the presence of a mutation and the sequencing results confirmed the presence of the mutations (see Table 5). In addition, there were no mutations detected by sequencing in samples scored as wild-type by HRM. This gave 100% sensitivity and 100% positive predictive value for HRM for the breast tumour sample set. Larger studies are needed to further assess the sensitivity and positive predictive value of the current methodology.

In the breast tumour sample set, sample B14 was interesting as it was scored as a query by HRM and subsequent sequencing showed the presence of a 531_533delC mutation that was present at a low percentage (Figure 8). Data from other HRM studies (our unpublished results) indicate that deletions/insertion of a single base do not have the same magnitude of change in melt profile compared to that of a heterozygous SNP. Another sample, B4 was interesting as the HRM data indicated a clear mutation but the sequencing data showed the mutation at the lower limit of detection. These cases reflected the lower proportion of tumour in some of the samples as there was no enrichment of the tumour component before DNA extraction.

One of the major advantages of HRM is for the detection of somatic mutations in genes that can have alterations at different positions in the coding sequence. Because in addition to pathogenic mutations, HRM will detect SNPs, silent mutations and intronic variation of unknown significance, and may have an intrinsic false positive rate, any aberrant melt curves should be validated by sequencing.

## Conclusion

This study validates the use of HRM as a method to scan for somatic mutations in *TP53*. The method is sensitive, rapid and cost effective and will markedly reduce the amount of sequencing required in mutational studies of *TP53 *and thereby the cost required for these studies.

## Competing interests

The author(s) declare that they have no competing interests.

## Authors' contributions

MK participated in primer design, carried out the molecular genetic studies and data analysis, and wrote the manuscript. AAA, SJH and JDB were responsible for conducting sequencing analysis of the ovarian cancer samples for *TP53 *mutations. DE was responsible for coordinating the samples, micro-dissection of the tumour material and preparation of the DNA. AdF provided ovarian tumour samples from the Westmead Gynaecological Oncology Tissue Bank. AOCS and the Westmead Gynaecological Oncology Tissue Bank  were responsible for collection, processing and storage of their respective samples. SJB provided the breast cancer DNA and contributed to the manuscript. DDB suggested the use of the sequenced ovarian cancer resource, was involved in the study design and contributed to the manuscript. AD originated the study, designed the primers, reviewed the data analysis and co-wrote the manuscript. All authors read and approved the final manuscript.

## Pre-publication history

The pre-publication history for this paper can be accessed here:



## Supplementary Material

Additional file 1 Difference plot of additional T47D dilutions. T47D was diluted at 95%, 90%, 75% and 1% (green) alongside original dilutions (orange). Wild type profiles are in blue. At dilutions of 95%, 90% and 75% the heteroduplex effect from the addition of wild-type DNA to T47D can be seen in altered shape of the melt profile. Click here for file
