# Supplementary figures and images for: High resolution melting for mutation scanning of TP53 exons 5–8
Source: BMC Cancer. 2007 Aug 31;7:168. doi: 10.1186/1471-2407-7-168 (PMC2025602; doi:10.1186/1471-2407-7-168)

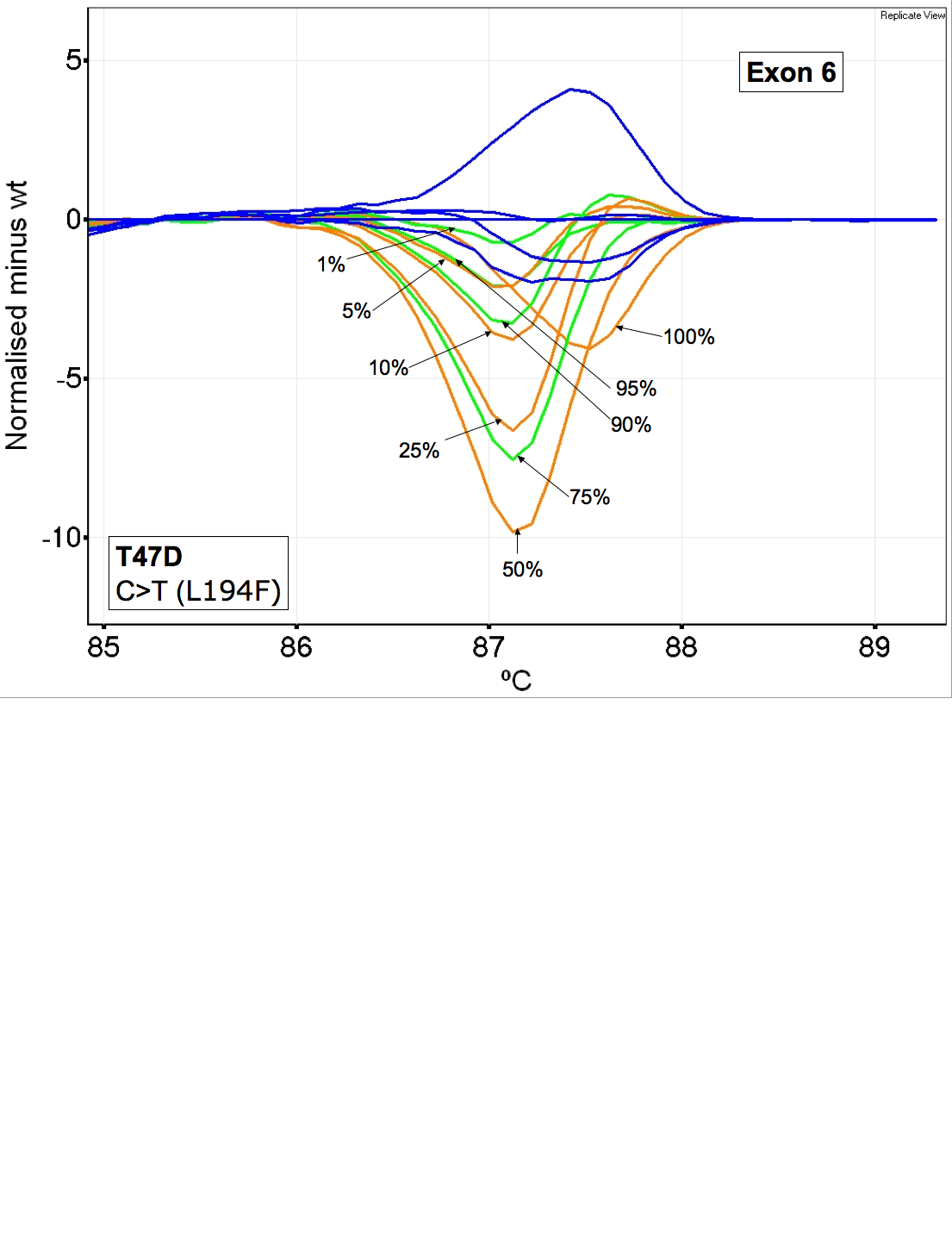

Supplement: Additional file 1 — Difference plot of additional T47D dilutions. T47D was diluted at 95%, 90%, 75% and 1% (green) alongside original dilutions (orange). Wild type profiles are in blue. At dilutions of 95%, 90% and 75% the heteroduplex effect from the addition of wild-type DNA to T47D can be seen in altered shape of the melt profile. [file 1471-2407-7-168-S1.png]
